# On the stability of a class of slowly varying systems

**DOI:** 10.1186/s13660-018-1934-1

**Published:** 2018-12-11

**Authors:** M. F. M. Naser, G. N. Gumah, S. K. Al-Omari, O. M. Bdair

**Affiliations:** Faculty of Engineering Technology, Al-Balqa Applied University, Amman, Jordan

**Keywords:** Slowly varying system, Stability, Lyapunov function

## Abstract

Slowly varying systems are common in physics and control engineering and thus stability analysis for those systems has drawn considerable attention in the literature. This paper uses the “frozen time approach” to derive Lyapunov inequality conditions for the stability of a wide class of slowly varying systems. These conditions refine those developed in (Khalil in Nonlinear Systems, 2002) and display generality and effectiveness for both linear and nonlinear systems. To illustrate the utility of the proposed results, an example has been included.

## Introduction

Slowly varying systems were first introduced in the 1960s by Desoer [5] in a one page article where he investigated conditions that ensure the exponential stability of an unforced linear system using the so-called “frozen time approach”. This approach draws conclusions on the stability of systems for any frozen time of an input function, a time-varying parameter or an internal/external disturbance. For instance, the input/output system $\dot{x}=f(x,u)$ is expected to possess stability results that are similar to the frozen system (i.e. when the input *u* is treated as a constant). Numerous techniques for solving slowly varying systems with parameters influenced by environmental conditions have been developed in [6, 9, 15, 18, 22]. The aforementioned parameters are typically smooth and involve sufficiently small derivatives; see [5], because otherwise the stability of the system is hard to guarantee [16].

Stability analysis of slowly varying systems can be simplified using the frozen time approach by approximating time-varying systems with slowly varying inputs or parameters by time-invariant ones. To this end, the system under study is required to be attractive or even asymptotically stable as well; see [4, 10, 14] and [20] for further details. This makes the Lyapunov analysis quite involved in studying such systems. For instance, [11] gives a method for constructing strict Lyapunov functions for the class of systems under study. Furthermore, the frozen parameter approach is used in the field of stabilizing feedback systems [8]. Alternatively, the stability of slowly varying systems can be described by eigenvalue-based methods as in [19].

Many references have been devoted to the study of the linear case as in [7] where a Popov criterion is given. The exponential stability and instability of continuous linear systems on time scales are studied in [2] and [3], respectively. In Ref. [21], the author investigates the stability conditions for certain continuous linear slowly varying system, while in Theorem 9.3 of [10], the author provides Lyapunov-based sufficient conditions for the stability of slowly varying systems in some detail.

The main contribution of this paper is new as far as we are aware. We establish a generalization of [10, Theorem 9.3] in a different perspective. We claim the generality, with additional implementations, that our results can be extended to both linear and nonlinear models and are highly suitable for the nonlinear case. To illustrate the described results, an example is given.

The present paper has the following structure. Section 2 presents some results and definitions that are used throughout the paper. In Sect. 3, we establish the main result of this article with the proposed conditions. Simulations are provided in Sect. 4. A brief conclusion part is added at the end of the paper.

## Background results and definitions

This section states some results and definitions that are needed in the paper.

### Lemma 2.1

([13, 14])

*Consider a function* $z:[t_{0},\omega)\rightarrow\mathbb{R}_{+}$ *where* $t_{0}\in\mathbb{R}$ *and* $t_{0}<\omega\leq\infty$. *Assume that*:

- *The function* *z* *is absolutely continuous on each compact interval of* $[t_{0},\omega)$.
- *There exist* $z_{1}\geq0$ *and* $z_{2}>0$ *such that* $z_{1}< z_{2}$, $z (t_{0} )< z_{2}$ *and* $\dot{z} (t )\leq0$ *for almost all* $t\in (t_{0},\omega )$ *that satisfy* $z_{1}< z (t )< z_{2}$.

*Then* $z (t )\leq\max{ (z (t_{0} ),z_{1} )}$, *for all* $t\in[t_{0},\omega)$.

### Lemma 2.2

([12, Corollary 2.6])

*Consider the differential equation*

1 $$ \dot{W} (t )=-\beta \bigl(W (t ) \bigr)+e (t ), $$

*where* $t\geq t_{0}$, $W(t)\in \mathbb{R}$, $\beta (\cdot )$ *is of class* $\mathcal{K}$ (*that is*, *continuous*, *strictly increasing*, *and* $\beta (0 )=0$), *and* $e (\cdot )$ *is a positive continuous function that goes to* 0 *as* $t\rightarrow\infty$. *Then each global solution* $W(t)$ *of Eq*. (1); *with a strictly positive initial value*, *goes to* 0 *as* $t\rightarrow\infty$.

### Definition 2.1

([1, p. 79])

The point $x=0$ of system $\dot{x} (t )=F (t,x (t ) )$, $x (t_{0} )=x_{0}$ is said to be:

(i) stable if for any $t_{0}\in\mathbb{R}_{+}$ and any $\varepsilon>0$, there is $c>0$ such that if $\vert x_{0} \vert < c$ then each solution *x* of $\dot{x} (t )=F (t,x (t ) )$, $x (t_{0} )=x_{0}$ is continuable on $[t_{0},\infty)$ and
  $$\bigl\vert x (t ) \bigr\vert < \varepsilon,\quad \mbox{for all } t\geq t_{0}, $$
(ii) uniformly stable if for any $\varepsilon>0$, there is $c>0$ such that, for each $t_{0}\in\mathbb{R}_{+}$ and each $\vert x_{0} \vert < c$, every solution *x* of system $\dot{x} (t )=F (t,x (t ) )$, $x (t_{0} )=x_{0}$ is continuable on $[t_{0},\infty)$ and
  $$\bigl\vert x (t ) \bigr\vert < \varepsilon,\quad \mbox{for all } t\geq t_{0}, $$
(iii) globally attractive if for all $t_{0}\in\mathbb{R}_{+}$ and all $x_{0}\in\mathbb{R}^{m}$, each solution *x* of system $\dot{x} (t )=F (t,x (t ) )$, $x (t_{0} )=x_{0}$ is continuable on $[t_{0},\infty)$ with $\lim_{t\rightarrow\infty}x (t )=0$,
(iv) globally asymptotically stable if it is stable and globally attractive.

## Main results

This section derives sufficient conditions for the stability of the following slowly varying system:

2 $$\begin{aligned} &\dot{x} (t ) = f \bigl(x (t ),u (t ) \bigr), \end{aligned}$$

3 $$\begin{aligned} &x (t_{0} ) = x_{0}, \end{aligned}$$

where $t\geq t_{0}$, solution $x (t )\in\mathbb{R}^{m}$, $f\in C^{0} (\mathbb{R}^{m}\times\mathbb{R}^{n},\mathbb{R}^{m} )$ with $f (0,0 )=0$, and $u\in\varGamma$ where $\varGamma= \{ u\in C^{1} (\mathbb{R},\mathbb{R}^{n} ): \exists \epsilon>0\mbox{ such that } \vert \dot{u} (\cdot ) \vert <\epsilon \} $; for some positive integers *m* and *n*. The origin $x=0$ is an equilibrium point for Eq. (2). One considers Eq. (2) to be slowly varying because all the elements of the set *Γ* are continuously differentiable with “sufficiently” small derivative (see [10]). The idea is to derive some stability properties that are uniformly valid in $u\in\varGamma$ (when *u* is treated as a frozen parameter).

Let $u\in\varGamma$, then the right-hand side of Eq. (2) is continuous. This implies that, for any $x_{0}\in\mathbb{R}^{m}$, Eq. (2) admits a continuous solution that is defined on a maximal interval of existence $[t_{0},\omega)$ where $\omega\in(t_{0},\infty]$. Moreover, each solution of Eq. (2) is continuously differentiable because of the continuity of the right-hand side of Eq. (2) (see [17]).

Theorem 9.3 in [10] studies the stability of the slowly varying system (2) under the assumptions (A1)–(A4):

(A1): There exists $h\in C^{1} (\mathbb{R}^{n},\mathbb{R}^{m} )$ such that $f (h (v ),v )=0$, for all $v\in\mathbb{R}^{n}$. Additionally, there exists some $L>0$ such that
  4 $$ \biggl\vert \frac{dh (v )}{dv} \biggr\vert _{2}\leq L, \quad \mbox{for all } v\in\mathbb{R}^{n}, $$
  where $\vert \cdot \vert _{2}$ is the induced 2-norm for matrices.
(A2): For the change of variables $y (\cdot )=x (\cdot )-h (u (\cdot ) )$, there exists a Lyapunov function $V_{*}\in C^{2} (\mathbb{R}^{m}\times\mathbb{R}^{n},\mathbb{R}_{+} )$ with a finite third derivative such that for all $\alpha_{1}\in\mathbb{R}^{m}$ and all $\alpha_{2}\in\mathbb{R}^{n}$ there exist some strictly positive numbers $c_{1}$, $c_{2}$, $c_{3}$, $c_{4}$, and $c_{5}$ satisfying
  5 $$\begin{aligned} &c_{1} \vert \alpha_{1} \vert ^{2}\leq V_{*} (\alpha_{1},\alpha_{2} )\leq c_{2} \vert \alpha_{1} \vert ^{2}, \end{aligned}$$
  6 $$\begin{aligned} &\biggl\vert \frac{\partial V_{*} (\alpha_{1},\alpha_{2} )}{\partial\alpha_{2}} \biggr\vert \leq c_{5} \vert \alpha_{1} \vert ^{2}, \end{aligned}$$
  7 $$\begin{aligned} &\biggl\vert \frac{\partial V_{*} (\alpha_{1},\alpha_{2} )}{\partial\alpha_{1}} \biggr\vert \leq c_{4} \vert \alpha_{1} \vert , \end{aligned}$$
  8 $$\begin{aligned} &\frac{\partial V_{*} (\alpha_{1},u (t ) )}{\partial\alpha_{1}} \biggm|_{\alpha_{1}=y (t )} {}\cdot f \bigl(y (t )+h \bigl(u (t ) \bigr),u (t ) \bigr)\leq-c_{3} \bigl\vert y (t ) \bigr\vert ^{2}, \end{aligned}$$
  for all $t\geq t_{0}$ and all $u\in\varGamma$.
(A3): The quantities *ϵ* and $|y(t_{0})|$ are less than some number that depends on *L* and $c_{i}$; $i=1,2,\ldots,5$.
(A4): One has $\lim_{t \to \infty} \dot{u}(t)=0$.

In the following theorem we relax Assumption (A2) of [10, Theorem 9.3] where we prove that the Lyapunov function $V_{*}$ needs only to be continuously differentiable (instead of being $C^{2}$ with a finite third derivative in [10, Theorem 9.3]). Moreover, in inequalities (5) and (8), we replace the functions $c_{i} \vert \cdot \vert ^{2}$, $i=1,2,3$, by class $\mathcal{K}_{\infty}$ functions (a continuous function *β* is of class $\mathcal{K}_{\infty}$ if it is strictly increasing with $\beta (0 )=0$ and $\lim_{t\rightarrow\infty}{\beta (t )}=\infty$). Furthermore, in inequalities (6) and (7), we replace the functions $c_{4} \vert \cdot \vert $ and $c_{5} \vert \cdot \vert ^{2}$ by a continuous function *H*.

### Theorem 3.1

*Suppose that*:

- *Assumption* (A1) *of* [10, *Theorem* 9.3] *is satisfied*.
- *For each solution* $x(t)$ *of the system* (2) *with maximal interval of existence* $[t_{0},\omega)$, *suppose that there exist* $\delta>0$, *a function* $V\in C^{1} (\mathbb{R}^{m}\times\mathbb{R}^{n},\mathbb{R}_{+} )$ *and class* $\mathcal{K}_{\infty}$ *functions* $\beta_{1}$, $\beta_{2}$ *and* $\beta_{3}$ *satisfying*
  9 $$\begin{aligned} &\beta_{1} \bigl( \vert \alpha_{1} \vert \bigr)\leq V (\alpha_{1},\alpha_{2} )\leq\beta_{2} \bigl( \vert \alpha_{1} \vert \bigr),\quad \textit{for all } ( \alpha_{1},\alpha_{2} )\in\mathbb{R}^{m}\times \mathbb{R}^{n}, \end{aligned}$$
  10 $$\begin{aligned} &\frac{\partial V (\alpha_{1},u (t ) )}{\partial\alpha_{1}} \biggm|_{\alpha_{1}=y (t )}{}\cdot f \bigl(y (t )+h \bigl(u (t ) \bigr), u (t ) \bigr)\leq-\beta_{3} \bigl( \bigl\vert y (t ) \bigr\vert \bigr), \end{aligned}$$
  *for all* $u\in\varGamma$ *and all* $t\in(t_{0},\omega)$ *that satisfy* $\vert y (t ) \vert <\delta$ *where* $y=x-h\circ u$.
- *There exists a nondecreasing function* $H\in C^{0} (\mathbb{R}_{+},\mathbb{R}_{+} )$ *such that* $H (v )>0$, *for all* $v>0$ *and*
  11 $$ \max{ \biggl( \biggl\vert \frac{\partial V (\alpha_{1},\alpha_{2} )}{\partial\alpha_{2}} \biggr\vert , \biggl\vert \frac{\partial V (\alpha_{1},\alpha_{2} )}{\partial\alpha_{1}} \biggr\vert \biggr)}\leq H \bigl( \vert \alpha_{1} \vert \bigr), $$
  *for all* $(\alpha_{1},\alpha_{2} )\in\mathbb{R}^{m}\times\mathbb{R}^{n}$ *that satisfy* $\vert \alpha_{1} \vert <\delta$.
- *One has* $\vert y (t_{0} ) \vert <\beta_{2}^{-1} (\beta_{1} (\delta ) )$ *and*
  12 $$ \epsilon< \frac{\beta_{3} (\beta_{2}^{-1} (\beta_{1} (\delta ) ) )}{ (L+1 )H (\delta )}. $$

*Then*:

(i) *For any* $u\in\varGamma$, *each solution* $x(t)$ *of the system* (2) *is continuable on* $[t_{0},\infty)$.
(ii) *If Assumption* (A4) *of* [10, *Theorem* 9.3] *is satisfied* (*that is*, $\dot{u} (t )\rightarrow 0$ *as* $t\rightarrow\infty$), *then we have* $\lim_{t\rightarrow\infty}y (t )=0$. (*This implies that* $\lim_{t\rightarrow\infty}x (t )=\lim_{t\rightarrow\infty}h (u (t ) )$ *whenever* $\lim_{t\rightarrow\infty}h (u (t ) )$ *exists*^1^).
(iii) *If* $h (\cdot )$ *is the zero function and* $V (\cdot,\cdot )$ *is independent of its second component* (*i*.*e*. *for every* $\alpha\in\mathbb{R}^{m}$, $V (\alpha,\cdot )$ *is a constant function*), *then*, *for any* $u\in\varGamma$, *the origin* $x=0$ *is uniformly stable and is globally asymptotically stable*.

We prove Results (i), (ii) and (iii) separately as follows.

*Proof of Result* (i): For any $u\in\varGamma$, let $x(t)$ be a solution of Eq. (2) with maximal interval of existence $[t_{0},\omega)$. For the change of variables $y (\cdot )=x (\cdot )-h (u (\cdot ) )$, we deduce by Eq. (2) that

13 $$ \dot{y} (t )=f \bigl(y (t )+h \bigl(u (t ) \bigr),u (t ) \bigr)- \frac{dh (v )}{dv} \biggm|_{v=u (t )}\dot{u} (t ),\quad t\geq t_{0}. $$

Define $z:[t_{0},\omega)\rightarrow\mathbb{R}_{+}$ as $z (t )=V (y (t ),u (t ) )$, for all $t\in[t_{0},\omega)$. Since all functions *x*, *h*, *u*, and *V* are continuously differentiable, the functions *y* and *z* are absolutely continuous on each compact interval of $[t_{0},\omega)$. One sees from (4), (10), (11), and (13) that, for all $t\in(t_{0},\omega)$ that satisfy $\vert y (t ) \vert <\delta$, one has

14 $$\begin{aligned} \dot{z} (t ) = & \frac{\partial V (\alpha_{1},u (t ) )}{\partial\alpha_{1}} \biggm|_{\alpha_{1}=y (t )}{}\cdot\dot{y} (t )+ \frac{\partial V (y (t ),\alpha_{2} )}{\partial\alpha_{2}} \biggm|_{\alpha_{2}=u (t )}{}\cdot\dot{u} (t ) \\ = & \frac{\partial V (\alpha_{1},u (t ) )}{\partial\alpha_{1}} \biggm|_{\alpha_{1}=y (t )}{}\cdot \biggl(f \bigl(y (t )+h \bigl(u (t ) \bigr),u (t ) \bigr) -\frac{dh (v )}{dv} \biggm|_{v=u (t )}\dot{u} (t ) \biggr) \\ & {}+\frac{\partial V (y (t ),\alpha_{2} )}{\partial\alpha_{2}} \biggm|_{\alpha_{2}=u (t )}{}\cdot\dot{u} (t ) \\ \leq & -\beta_{3} \bigl( \bigl\vert y (t ) \bigr\vert \bigr)+ (L+1 )H \bigl( \bigl\vert y (t ) \bigr\vert \bigr) \bigl\vert \dot{u} (t ) \bigr\vert . \end{aligned}$$

### Claim 1

$\omega=\infty$ *and* $\Vert y \Vert _{\infty}<\infty$.

### Proof

Since *H* is nondecreasing we conclude from inequality (14) that

15 $$ \begin{aligned}[b] &\dot{z} (t )\leq -\beta_{3} \bigl( \bigl\vert y (t ) \bigr\vert \bigr)+ (L+1 )H (\delta ) \bigl\vert \dot{u} (t ) \bigr\vert \\ &\quad \mbox{for all } t\in(t_{0},\omega), \mbox{ that satisfy } \bigl\vert y (t ) \bigr\vert < \delta. \end{aligned} $$

We get by inequality (12) that $\beta_{3}^{-1} ( (L+1 )H (\delta )\epsilon )<\beta_{2}^{-1} (\beta_{1} (\delta ) )$ and hence (9) implies that $\beta_{3}^{-1} ( (L+1 )H (\delta )\epsilon )<\delta$. Thus the fact that $\vert \dot{u} (\cdot ) \vert <\epsilon$ and (15) lead to

16 $$ \dot{z} (t )\leq 0, \quad \mbox{for all } t \in(t_{0},\omega),\mbox{ that satisfy } \beta_{3}^{-1} \bigl( (L+1 )H (\delta )\epsilon \bigr)< \bigl\vert y (t ) \bigr\vert < \delta. $$

Inequality (12) implies that $\beta_{2} (\beta_{3}^{-1} ( (L+1 )H (\delta )\epsilon ) )<\beta_{1} (\delta )$. Hence, we deduce by inequalities (9) and (16) that

17 $$ \dot{z} (t )\leq 0, \quad \mbox{for all }t \in(t_{0},\omega),\mbox{ that satisfy } \beta_{2} \bigl(\beta_{3}^{-1} \bigl( (L+1 )H (\delta )\epsilon \bigr) \bigr)< z (t )< \beta_{1} (\delta ). $$

On the other hand, since it is assumed that $\vert y (t_{0} ) \vert <\beta_{2}^{-1} (\beta_{1} (\delta ) )$, we obtain by inequality (9) that $z (t_{0} )<\beta_{1} (\delta )$. Therefore, we deduce by (17) that all conditions of Lemma 2.1 are satisfied with $z_{2}=\beta_{1} (\delta )$ and $z_{1}=\beta_{2} (\beta_{3}^{-1} ( (L+1 )H (\delta )\epsilon ) )$. Hence

$$z (t )\leq \max{ \bigl(\beta_{2} \bigl(\beta_{3}^{-1} \bigl( (L+1 )H (\delta )\epsilon \bigr) \bigr),z (t_{0} ) \bigr)},\quad \mbox{for all } t\in[t_{0},\omega). $$

Thus (9) leads to $\vert y (t ) \vert \leq M$, for all $t\in[t_{0},\omega)$ where

$$M=\max{ \bigl(\beta_{1}^{-1} \bigl(\beta_{2} \bigl( \beta_{3}^{-1} \bigl( (L+1 )H (\delta )\epsilon \bigr) \bigr) \bigr),\beta_{1}^{-1} \bigl(\beta_{2} \bigl( \bigl\vert y(t_{0}) \bigr\vert \bigr) \bigr) \bigr)}. $$

This implies that $\Vert y \Vert _{\infty}<\infty$ so that $\omega=\infty$, which completes the proof of the claim. □

Claim 1 proves that each solution $x(t)$ of the system (2) is continuable on $[t_{0},\infty)$, which completes the proof of Result (i).

*Proof of Result* (ii): Assume that $\lim_{t\rightarrow\infty}\dot{u} (t )=0$. Since *H* is nondecreasing, we deduce by Claim 1 and inequalities (9) and (14) that

$$\dot{z} (t )\leq-\beta \bigl(z (t ) \bigr)+e (t ),\quad \mbox{for all }t>t_{0}, $$

where $\beta (\cdot )=\beta_{3}\circ\beta_{2}^{-1} (\cdot )$ and $e (\cdot )= (L+1 )H (M ) \vert \dot{u} (\cdot ) \vert $. The function $\beta (\cdot )$ is of class $\mathcal{K}_{\infty}$. Moreover, since $\lim_{t\rightarrow\infty} \vert \dot{u} (t ) \vert =0$, one has $\lim_{t\rightarrow\infty}e (t )=0$. Consider the differential equation

18 $$\begin{aligned} &\dot{W} (t ) = -\beta \bigl(W (t ) \bigr)+e (t ), \end{aligned}$$

19 $$\begin{aligned} &W(t_{0}) = z(t_{0}), \end{aligned}$$

where $t\geq t_{0}$, $W (t )\in\mathbb{R}$. Due to the continuity of the right-hand side of Eq. (18), a continuous solution for the system (18)–(19) exists and is defined on a maximal interval of existence $[t_{0},t_{1})$ where $t_{0}< t_{1}\leq\infty$. We observe that the initial state $W(t_{0})=z(t_{0})$ is nonnegative by the definition of the function *z*.

### Claim 2

*Each solution* $W (\cdot )$ *of the system* (18)*–*(19) *is nonnegative*, *continuable on* $[t_{0},\infty)$, *and satisfies* $\lim_{t\rightarrow\infty}{W (t )}=0$.

### Proof

Assume that there is some $t_{2}\in[t_{0},t_{1})$ such that $W(t_{2})<0$. The continuity of *W* implies that it attains its infimum on the compact interval $[t_{0},t_{2}]$; say $\inf_{t\in[t_{0},t_{2}]}{W (t )}=W (t_{3} )$ for some $t_{3}\in (t_{0},t_{2}]$. Hence $W (t_{3} )<0$ and $\dot{W} (t_{3} )=0$. Thus Eq. (18) leads to $0=\dot{W} (t_{3} )=-\beta (W (t_{3} ) )+e (t )>0$. This contradiction proves that $W (\cdot )$ is nonnegative. On the other hand, the fact that $\lim_{t\rightarrow\infty}e (t )=0$ leads to $\Vert e \Vert _{\infty}<\infty$ and $\dot{W} (t )\leq-\beta (W (t ) )+ \Vert e \Vert _{\infty}$, for all $t\geq t_{0}$. This implies that $\dot{W} (t )\leq0$, for all $t\geq t_{0}\mbox{ that satisfy }W (t )>\beta^{-1} ( \Vert e \Vert _{\infty} )$. Therefore, all conditions of Lemma 2.1 are satisfied with $z_{1}=\beta^{-1} ( \Vert e \Vert _{\infty} )$ and $z_{2}=W(t_{0})+1$. Thus we get $W (t )\leq\max{ \{ W(t_{0}),\beta^{-1} ( \Vert e \Vert _{\infty} ) \} }$. The boundedness of $W(t)$ implies that each solution of the system (18)–(19) is continuable on $[t_{0},\infty)$. Now we need to show that $\lim_{t\rightarrow\infty}{W (t )}=0$. To this end, consider the following cases.

(i) If $W(t_{0})>0$, the property $\lim_{t\rightarrow\infty}{W (t )}=0$ follows from Lemma 2.2.
(ii) If $W(t_{0})=0$, then either *W* is the zero function or can be strictly positive at some element in its domain. When *z* is the zero function, the property $\lim_{t\rightarrow\infty}{W (t )}=0$ is trivially valid. Otherwise, there exists some $t_{4}>t_{0}$ such that $z(t_{4})>0$; then, by seeing the number $t_{4}$ as a new initial time, one can simply deduce by Lemma 2.2 that $\lim_{t\rightarrow\infty}{W (t )}=0$. This completes the proof of the claim.

 □

By Claim 2, one can use the comparison lemma [10, p. 102] to deduce that $z (t )\leq W (t )$, for all $t\geq t_{0}$. Hence the fact that $\lim_{t\rightarrow\infty}W (t )=0$ leads to $\lim_{t\rightarrow\infty}{z (t )}=0$. Thus inequality (9) implies that $\lim_{t\rightarrow\infty}y (t )=0$ and Result (ii) is seen to be true.

*Proof of Result* (iii): Assume that $h (\cdot )$ is the zero function and that $V (\cdot,\cdot )$ is independent of its second component. Then we have $x (\cdot )=y (\cdot )$. We have by inequality (10) that

20 $$ \frac{dV (\alpha )}{d\alpha} \biggm|_{\alpha=x (t )}{}\cdot f \bigl(x (t ),u (t ) \bigr)\leq-\beta_{3} \bigl( \bigl\vert x (t ) \bigr\vert \bigr),\quad \mbox{for all } t\geq t_{0}. $$

This makes $V (\cdot )$ a strict Lyapunov function [1, Theorem 3.2] which implies that the origin $x=0$ is uniformly stable and is globally asymptotically stable.

### Corollary 3.1

*Suppose that all assumptions of Theorem* 3.1 *are satisfied*. *If* $\lim_{t\rightarrow\infty}{u(t)}=u_{*}$ *exists finitely*, *then*

$$\lim_{t\rightarrow\infty}{\dot{x}(t)}=f \bigl(h (u_{*} ),u_{*} \bigr). $$

### Proof

It follows by Eq. (2), the result $\lim_{t\rightarrow\infty} (x (t )-h (u (t ) ) )=0$ of Theorem 3.1 and the continuity of the function *h*. □

We observe that the special cases $\beta_{i} (\cdot )=c_{i} \vert \cdot \vert ^{2}$, $i=1,2,3$, and $H (\cdot )=\max{ (c_{4} \vert \cdot \vert ^{2},c_{5} \vert \cdot \vert )}$ makes Items (b) and (c) of Theorem 3.1 reduced to Assumption (A2) of [10, Theorem 9.3]. Moreover, Assumptions (A1), (A3) and (A4) of [10, Theorem 9.3] are already assumed in Theorem 3.1 (see Items (a) and (d)).

## Example and simulations

This section uses Theorem 3.1 to study the stability of a nonlinear slowly varying system without any restriction on the initial conditions or the magnitude of the function *u̇*. Consider the nonlinear system

21 $$\begin{aligned} \dot{x}_{1} (t ) = & -x_{1}^{3} (t )+x_{2} (t )-\mu\sin{ \bigl(u (t ) \bigr)}, \end{aligned}$$

22 $$\begin{aligned} \dot{x}_{2} (t ) = & -x_{1} (t )- \bigl(2x_{1}^{2} (t )+1 \bigr) \bigl(x_{2} (t )-\mu\sin{ \bigl(u (t ) \bigr)} \bigr), \end{aligned}$$

where $\mu>0$, $t\geq t_{0}$, $u\in C^{1} (\mathbb{R},\mathbb{R} )$ and $x(t)=\left[\begin{array}{c} x_{1}(t)\\ x_{2}(t) \end{array}\right]$ takes values in $\mathbb{R}^{2}$. The system (21)–(22) has the form of (2) where $n=1$, $m=2$ and

$$g(\alpha ,v)=\left[\begin{array}{c} -\alpha_{*}^{3}+\alpha_{**}-\mu \sin(v)\\ -\alpha_{*}-(2\alpha_{*}^{2}+1)(\alpha_{**}-\mu \sin(v)) \end{array}\right],$$

for all $\alpha =\left[\begin{array}{c} \alpha_{*}\\ \alpha_{**} \end{array}\right]\in R^{2}$ and $v\in\mathbb{R}$. The continuously differentiable function $h(v)=\left[\begin{array}{c} 0\\ \mu \sin(v) \end{array}\right]$, for all $v\in\mathbb{R,}$ satisfies the equality $f (h (v ),v )=0$, for all $v\in\mathbb{R}$. Since $\frac{dh(v)}{dv}=\left[\begin{array}{c} 0\\ \mu \cos(v) \end{array}\right]$, for all $v\in\mathbb{R}$, inequality (4) is satisfied with $L=\mu$.

Let $u\in\varGamma$ and let $x(t)$ be a solution of the system (21)–(22) with maximal interval of the form $[t_{0},\omega)$. Consider the Lyapunov function $V\in C^{1} (\mathbb{R}^{2},\mathbb{R}_{+} )$ that is defined as

$$V(\alpha )=\frac{1}{2}\left(\alpha_{*}^{2}+\alpha_{**}^{2}\right)=\frac{1}{2}|\alpha {|}^{2},\;\text{for all }\alpha =\left[\begin{array}{c} \alpha_{*}\\ \alpha_{**} \end{array}\right]\in R^{2}.$$

Inequality (9) is satisfied with $\beta_{1} (\cdot )=\beta_{2} (\cdot )=\frac{1}{2} (\cdot )^{2}$. Moreover, one has

$$\frac{dV(\alpha )}{d\alpha }=\left[\begin{array}{cc} \frac{dV(\alpha )}{d\alpha_{*}} & \frac{dV(\alpha )}{d\alpha_{**}} \end{array}\right]=\left[\begin{array}{cc} \alpha_{*} & \alpha_{**} \end{array}\right],\;\text{for all }\alpha =\left[\begin{array}{c} \alpha_{*}\\ \alpha_{**} \end{array}\right]\in R^{2}.$$

This implies that inequality (11) is satisfied with *H* being the identity function.

Consider the change of variables $y(\cdot )=\left[\begin{array}{c} y_{1}(\cdot )\\ y_{2}(\cdot ) \end{array}\right]=x(\cdot )-h(u(\cdot ))$. Consider an initial condition $x_{0}\in\mathbb{R}^{2}$ and a positive constant *ϵ*. Let *δ* be a positive constant such that

23 $$ \delta>\max{ \bigl(1, \bigl\vert y (t_{0} ) \bigr\vert , \epsilon (\mu+1 ) \bigr)}. $$

Hence $\vert y (t_{0} ) \vert <\beta_{2}^{-1} (\beta_{1} (\delta ) )=\delta$ and $\delta>1$. We conclude by Eqs. (21) and (22) that, for all $t\in[t_{0},\omega)$ that satisfy $\vert y (t ) \vert <\delta$, one has

$$\begin{aligned} & \frac{d V (\alpha )}{d\alpha} \biggm|_{\alpha=y (t )}{}\cdot g \bigl(y (t )+h \bigl(u (t ) \bigr),u (t ) \bigr) \\ &\quad = -y_{1}^{4} (t )-2y_{1}^{2} (t )y_{2}^{2} (t )-y_{2}^{2} (t ) \\ &\quad \leq -y_{1}^{4} (t )-2y_{1}^{2} (t )y_{2}^{2} (t )-\frac{1}{\delta^{2}}y_{2}^{4} (t ) \\ &\quad \leq -\frac{1}{\delta^{2}} \bigl(y_{1}^{4} (t )+2y_{1}^{2} (t )y_{2}^{2} (t )+y_{2}^{4} (t ) \bigr) \\ &\quad = -\frac{1}{\delta^{2}} \bigl(y_{1}^{2} (t )+y_{2}^{2} (t ) \bigr)^{2}=-\frac{1}{\delta^{2}} \bigl\vert y (t ) \bigr\vert ^{4}. \end{aligned}$$

Thus inequality (10) is satisfied with $\beta_{3} (\cdot )=\frac{1}{\delta^{2}} (\cdot )^{4}$. By inequality (23) we have $\delta>\epsilon (\mu+1 )$. Hence inequality (12) is satisfied. All conditions of Theorem 3.1 are satisfied. Therefore for every $x_{0}\in\mathbb{R}^{2}$ and $\epsilon>0$, each solution of the system (21)–(22) is continuable on $[t_{0},\infty)$. Moreover, if $\lim_{t\rightarrow\infty}\dot{u} (t )=0$, we have $\lim_{t\rightarrow\infty}y (t )=0$. This implies that $\lim_{t\rightarrow\infty}x_{1} (t )=0$ and $\lim_{t\rightarrow\infty} (x_{2} (t )-\mu\sin{ (u (t ) )} )=0$. As a result, if $\lim_{t\rightarrow\infty}{u(t)}$ exists finitely, then Corollary 3.1 and Eq. (21) lead to $\lim_{t\rightarrow\infty}{\dot{x}(t)}=0$.

In this example, we have proved that the quadratic Lyapunov function satisfies all conditions of Theorem 3.1 with $\beta_{3} (\cdot )=\frac{1}{\delta^{2}} (\cdot )^{4}$ (i.e. $\nabla V\cdot f\leq-\beta_{3} ( \vert y \vert )=-\frac{1}{\delta^{2}} \vert y \vert ^{4}$ for small $\vert y \vert $). This implies that there is no guarantee on the inequality $\nabla V\cdot f\leq-c_{3} \vert y \vert ^{2}$ to be valid for small $\vert y \vert $ and so is inequality (8).

**Figure 1:**
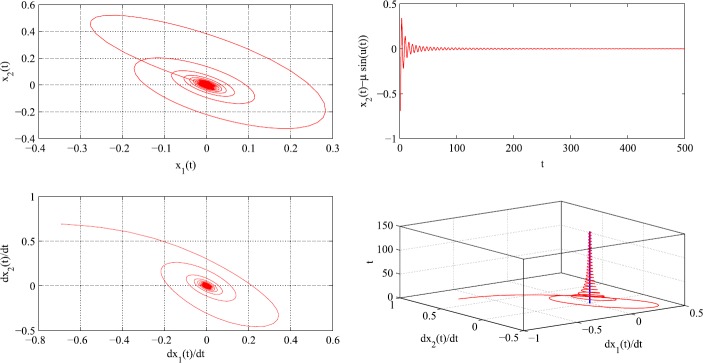
Simulations

*Simulations*: Let $t_{0}=1$, $\mu=1$, $x_{0}=\left[\begin{array}{c} 0\\ 0 \end{array}\right]$ and $u (t )=\frac{\sin{ (t )}}{t+0.1}$, for all $t\geq0$. We have $\lim_{t\rightarrow\infty}u (t )=\lim_{t\rightarrow\infty}\dot{u} (t )=0$. Thus, $\lim_{t\rightarrow\infty}x_{2} (t )=\lim_{t\rightarrow\infty}\mu\sin{ (u (t ) )}=0$ (see Fig. 1 (top-right)) and $\lim_{t\rightarrow\infty}x_{1} (t )=0$. This is also illustrated in Fig. 1 (top-left) which shows the plot of $x_{2}(t)$ versus $x_{1}(t)$ and clarifies that $(0,0)$ is the starting and the limiting point in the $x_{1} x_{2}$-plane. Since $\lim_{t\rightarrow\infty}{u(t)}$ exists finitely, one has $\lim_{t\rightarrow\infty}{\dot{x}_{1}(t)}=\lim_{t\rightarrow\infty}{\dot{x}_{2}(t)}=0$, which is clarified in Fig. 1 (bottom-left). In Fig. 1 (bottom-right), we observe that as $t\rightarrow\infty$ the red parametric curve *t* versus $\dot{x}_{2}(t)$ versus $\dot{x}_{1}(t)$ converges to the blue line which is parallel to the *t* axis and passes through $(0,0)$ in the $\dot{x}_{1}\dot{x}_{2}$-plane. This ensures the property $\lim_{t\rightarrow\infty}{\dot{x}_{1}(t)}=\lim_{t\rightarrow\infty}{\dot{x}_{2}(t)}=0$.

## Conclusion

We have provided sufficient conditions that ensure the stability of the slowly varying system $\dot{x} (t )=f (x (t ),u(t) )$ where *u* is treated as a “frozen parameter”. These conditions open the routes to further knowledge on the stability of more generic classes of systems. Numerical simulations for the nonlinear case have been carried out to illustrate the results.

## Footnotes

1 Observe that the converse of Barbalat’s lemma is not true; that is, the fact that $\lim_{t\rightarrow\infty}\dot{u} (t )=0$ does not necessarily imply that $\lim_{t\rightarrow\infty}u (t )$ exists. To see this take $t_{0}$ positive, $n=1$ and for all $t\geq t_{0}$ one has $u (t )=\sin{ (\ln{t} )}$.
